# Supplementary material for: Legumain Functions as a Transient TrkB Sheddase
Source: Int J Mol Sci. 2023 Mar 11;24(6):5394. doi: 10.3390/ijms24065394 (PMC10049731; doi:10.3390/ijms24065394)
Supplement: Supplementary file 1 [file ijms-24-05394-s001.zip › ijms-2247533-supplementary.pdf]

Figure S1: Posttranslational modifications of rTrkB-Ig2, Purification of neurotrophins and disulfide analysis of BDNF.

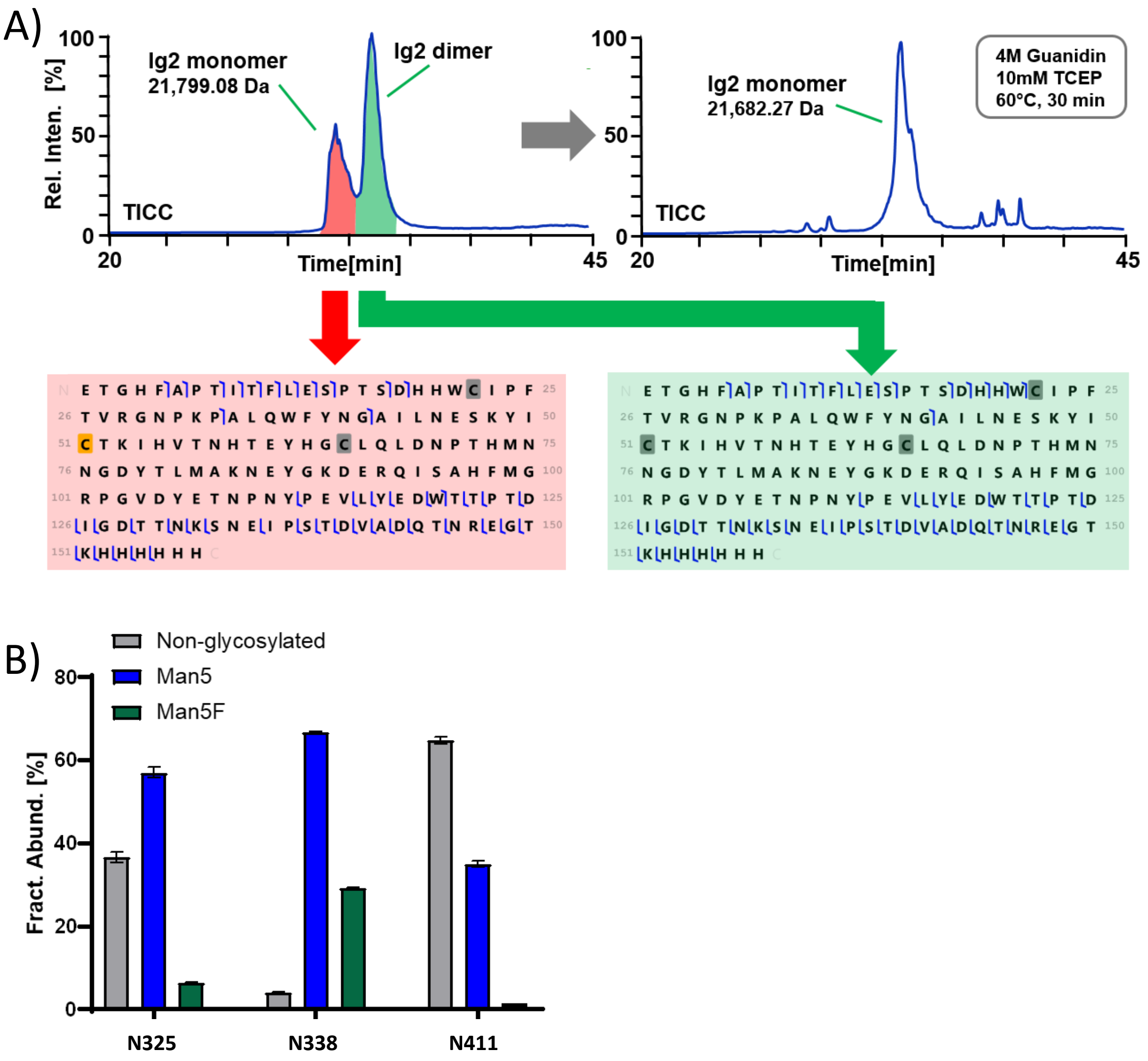

A: Total ion current chromatograms (TICC) of intact and reduced TrkB Ig2. Intact TrkB Ig2 revealed two major peaks that were identified as cysteinylated monomer (red) and dimer (green). After denaturation and reduction, only the non-cysteinylated monomer was observable. Assignment of MS^2 fragments generated from Ig2 monomer (red) and dimer (green). y- and b-ions were matched to the Ig2 sequence for both chromatographic peaks (blue lines).

B: Site-specific *N*-glycosylation of TrkB Ig2. Sites, N325, N338 and N411, were either not glycosylated or occupied by Man5 or its fucosylated counterpart. Data was obtained from MS^1-based quantification.

Figure S1: Posttranslational modifications of rTrkB-Ig2, Purification of neurotrophins and disulfide analysis of BDNF.

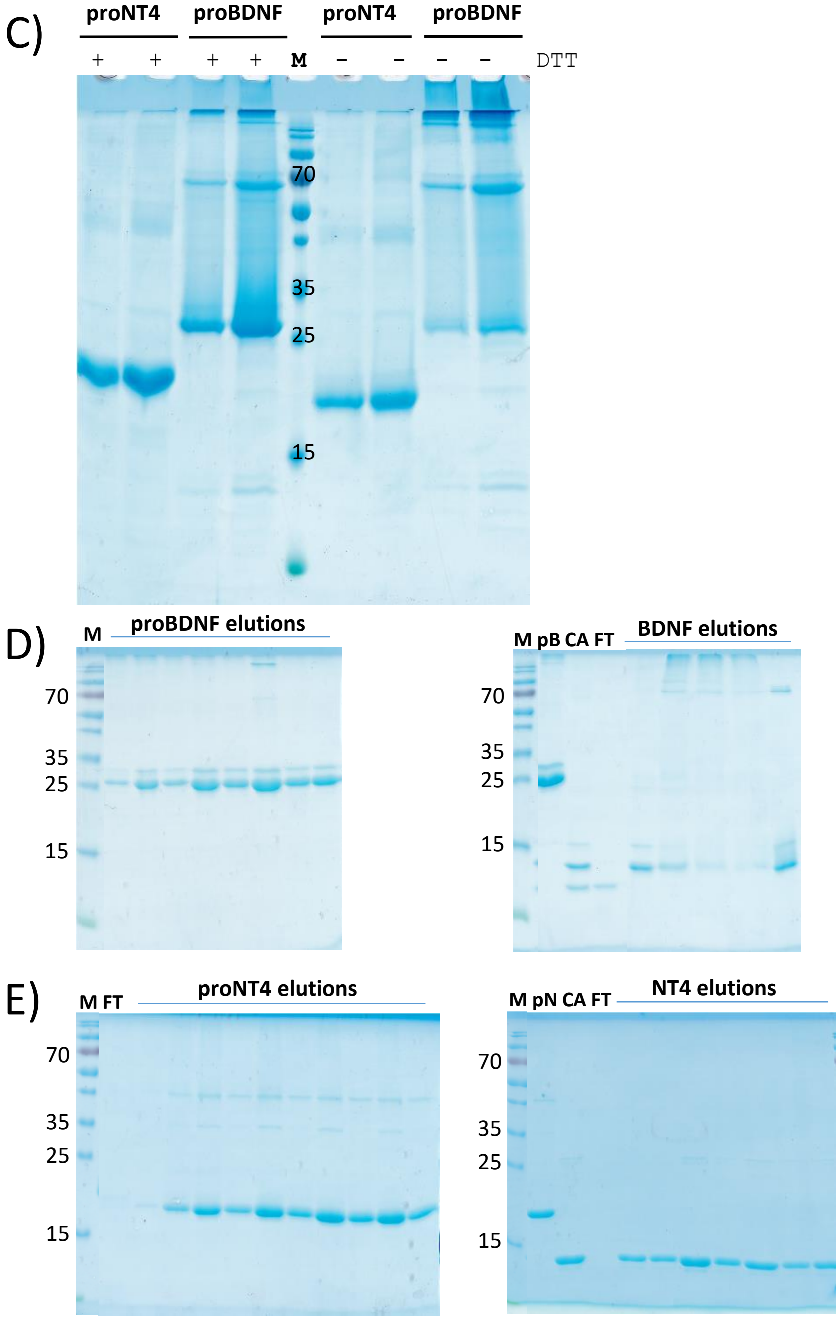

Purification steps of the recombinant neurotrophins.

C: Recombinant neurotrophins after folding.

D: proBDNF and BDNF after purification and activation, respectively.

E: As a proof of concept, we confirmed that the in vitro folding and activation protocols could, with minor adaptions, be also applied to proNT4 and NT4. proNT4 was purified on Ni-NTA rather than an SP-Sepharose in case of proBDNF.

Figure S1: Posttranslational modifications of rTrkB-Ig2, Purification of neurotrophins and disulfide analysis of BDNF.

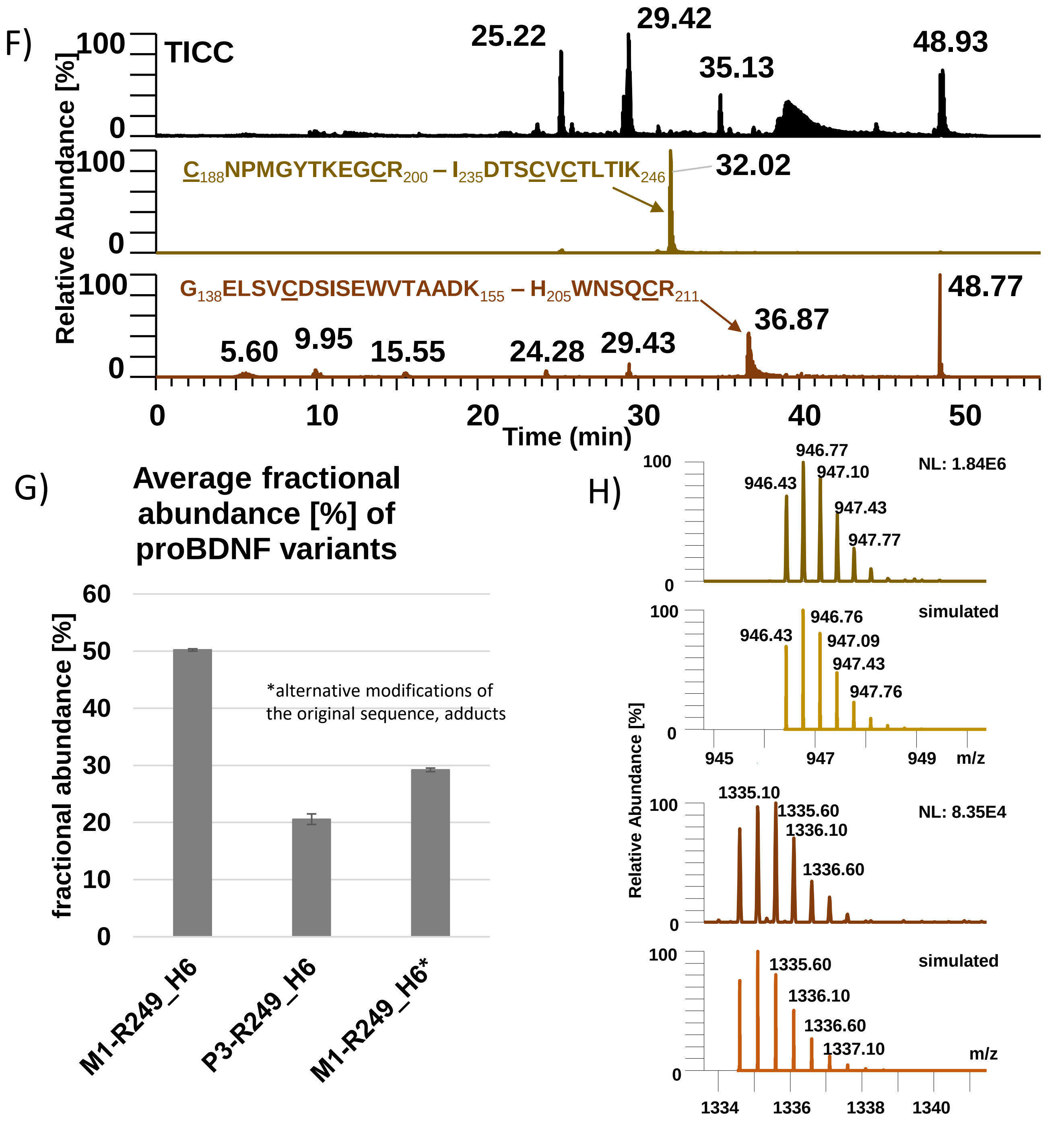

F: Total ion current (TIC) c Purification steps of the recombinant neurotrophins. hromatogram of tryptic peptides of proBDNF (top, black). Below extracted ion chromatograms for C_{188}NPMGYTKEGCR$_{200}$ – I_{235}DTSCVCTLTIK$_{246}$ (middle, yellow) and C_{188}NPMGYTKEGCR$_{200}$ – I_{235}DTSCVCTLTIK$_{246}$ (bottom, brick) disulfide linked peptides are shown.

G: Deconvolution to a zero-charge mass spectrum revealed three major proBDNF variants: M1-R246_H6 (of mass 27,230.96 Da with a fractional abundance of 50.23%), P3-R249_H6 (of mass 27,028.89 Da with a fractional abundance of 20.55%), and M1-R249_H6 (of masses slightly higher than 27,230.96 Da with a fractional abundance of 29.22%).

H: Extraction of mass spectra resulting of the Cys^{143}-Cys^{210} linked peptide (upper, yellow, charge state 3+) and the Cys^{188}-Cys^{239} and Cys^{199}-Cys^{241} linked dipeptide (bottom, brick, charge state 2+) revealed presence of the corresponding ions. The upper mass spectrum always represents the measured peptide, the lower depicts the simulated one.

Figure S2: Conserved legumain cleavage sites in TrkB and the impact of cleavage on TrkB structural integrity.

```
Q91987|NTRK2_CHICK              360  LLAKNEYGEDEKRVDAHFMSVPG-DGSGPIVDPD--VYEYETTPNDLGDTTNNSNQITSP  416
P15209|NTRK2_MOUSE              361  LMAKNEYGKDERQISAHFMGRPGVDYETNPNYPEVLYEDWTT-PTDIGDTTNKSNEIPST  419
Q63604|NTRK2_RAT                361  LMAKNEYGKDERQISAHFMGRPGVDYETNPNYPEVLYEDWTT-PTDIGDTTNKSNEIPST  419
Q16620|NTRK2_HUMAN              361  LIAKNEYGKDEKQISAHFMGWPGIDDGANPNYPDVIYEDYGTAANDIGDTTNRSNEIPST  420
A0A2J8MRU2|A0A2J8MRU2_PANTR     361  LIAKNEYGKDEKQISAHFMGWPGIDDGANPNYPDVIYEDYGTAANDIGDTTNRSNEIPST  420
E1BCQ4|E1BCQ4_BOVIN             361  LVAKNEYGKDEKQISAHFMGWPGIDDGANPNYPDVIYEDYGTAANDIGDTTNKSNGTLPT  420
A0A287A6X2|A0A287A6X2_PIG       361  LVAKNEYGKDEKQISAHFMGWPGIDDGANPNYPDVIYEDYGTAANDIGDTTNRSNEIPST  420
```

A: Amino acid sequence alignment of TrkB membrane linker indifferent species (chicken, mouse, rat, human, chimp, bovine, pig), revealing the strict conservation of the legumain cleavage sites in mammals.

B)

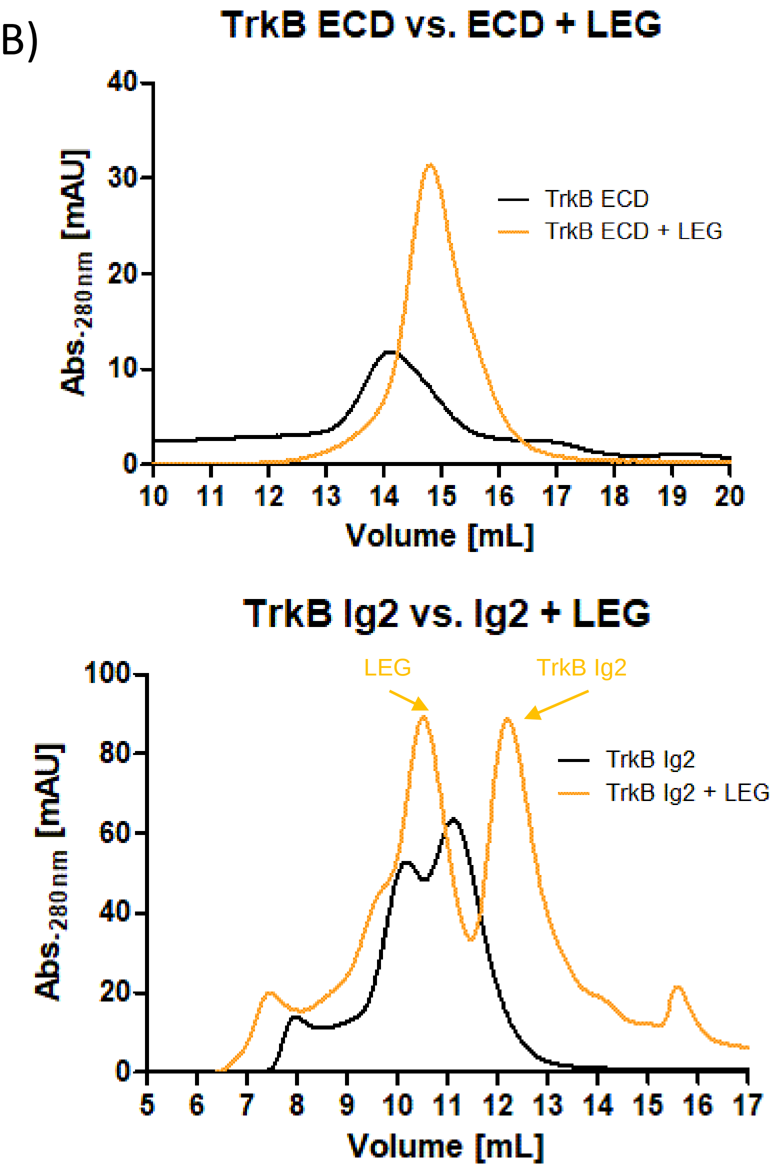

C)

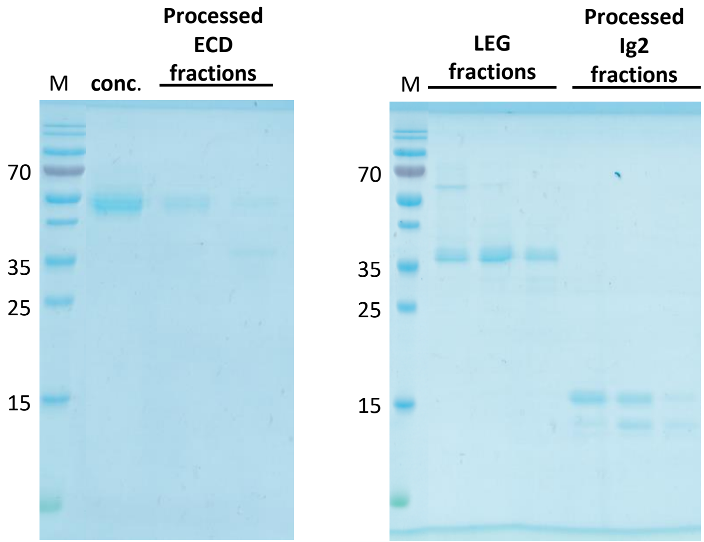

Figure S2: Conserved legumain ("LEG") cleavage sites in TrkB and the impact of cleavage on TrkB structural integrity.

B: Gel filtration runs of untreated and legumain ("LEG")-processed TrkB ECD and Ig2. In the ECD chromatogram, the untreated and processed TrkB ECD domains elute at 14 ml and 15 ml, respectively, reflecting the release of the flexible domain linker peptide. Of note, both TrkB ECD and legumain elute in a single peak at 15 ml. In the legumain-processed TrkB Ig2 chromatogram, legumain (left peak, orange) and processed Ig2 (right peak, orange) elute in clearly separated peaks at 10.5 ml and 12.5 ml, respectively. The unprocessed Ig2 domain elutes in a broad double peak at around 10 - 11 ml, which may reflect a weak homo-dimerization tendency.

C: SDS-PAGEs of pooled and concentrated TrkB ECD and Ig2 fractions.

Figure S3: pH-dependency of Legumain activity and proBDNF appears to partially prevent TrkB cleavage by legumain.

A)

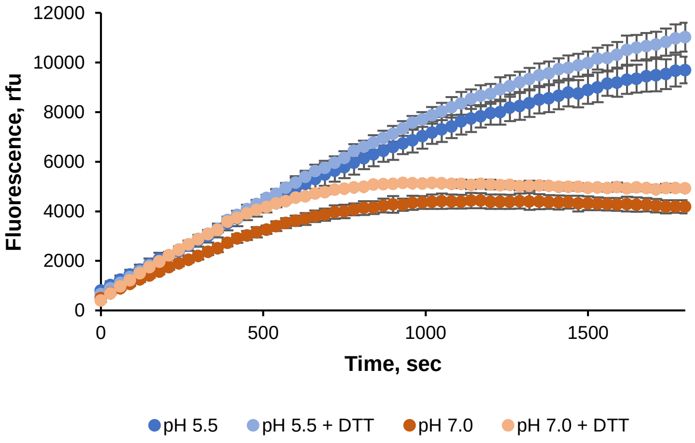

B)

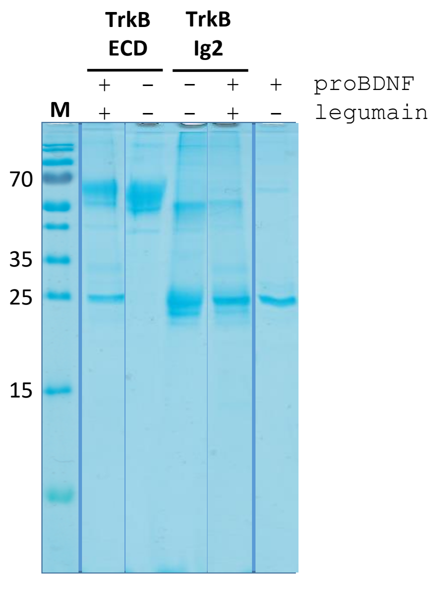

A: Turnover of the legumain specific substrate Z-Ala-Ala-Asn-AMC was measured at pH 5.5 and 7.0 in the presence and absence of the reducing agent DTT (5 mM).

B: In presence of legumain (+) and proBDNF (+) the intensities of the TrkB ECD (~68 kDa) and TrkB Ig2 (~25 kDa) bands appear reduced, indicating a partial processing by legumain.

Figure S4: Complex binding behavior of TrkB with BDNF

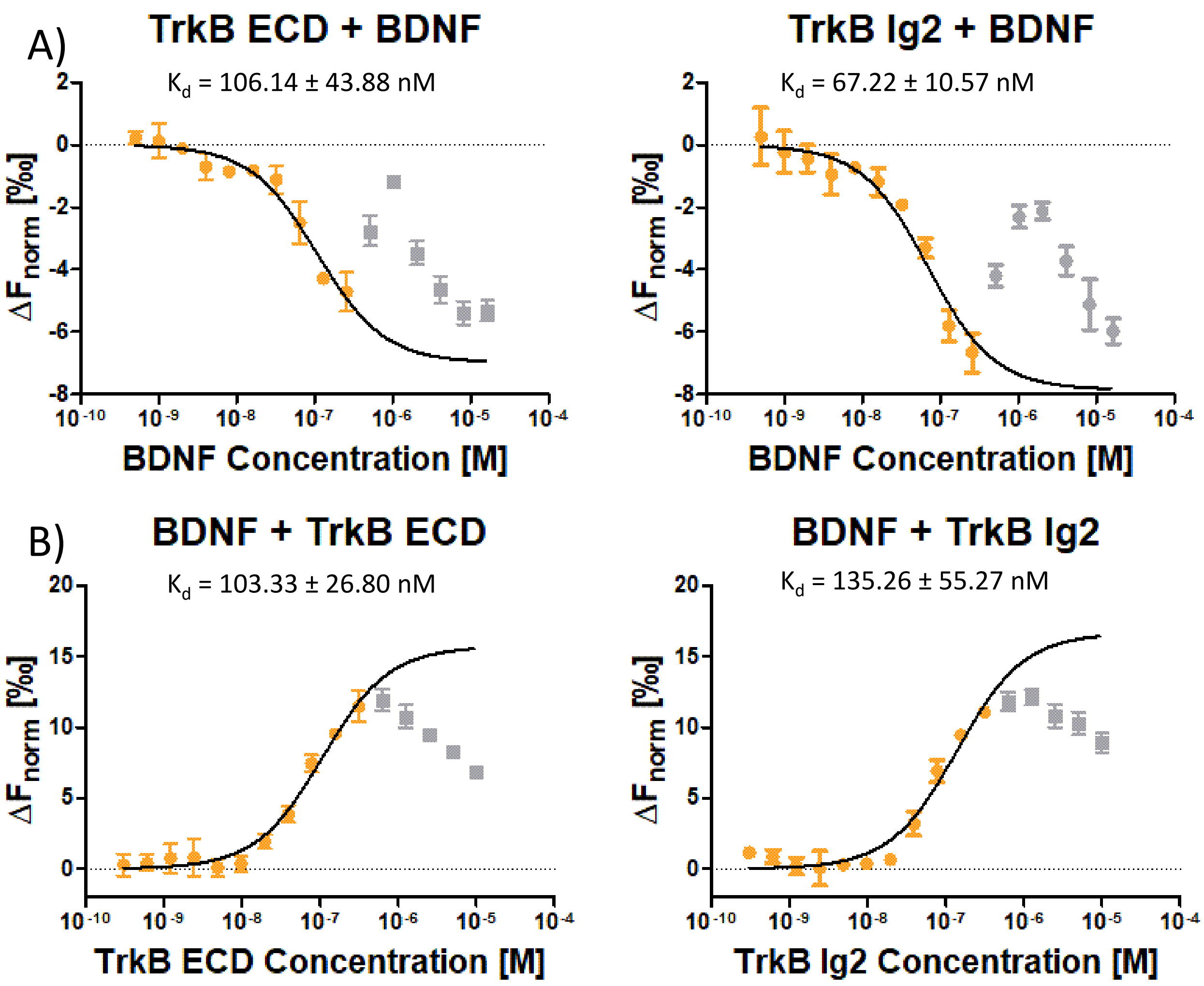

A: In a first experimental setup, fluorescently labeled TrkB variants (ECD, Ig2) were kept at constant concentration and titrated against increasing BDNF concentrations. In this thermophoresis experiments, TrkB ECD bound BDNF in solution with a K_d of 106 nM and TrkB Ig2 bound BDNF in solution with a K_d of 67 nM.

B: In a second setup, fluorescently labeled BDNF was kept at constant concentration. This setup resulted in dissociation constants K_d = 103 nM for TrkB ECD with BDNF and K_d = 135 nM for TrkB Ig2 with BDNF. Of note, in all four cases secondary binding events were observed, as discussed in the main text and Fig. S4C.

Figure S4: Complex binding behavior of TrkB with BDNF.

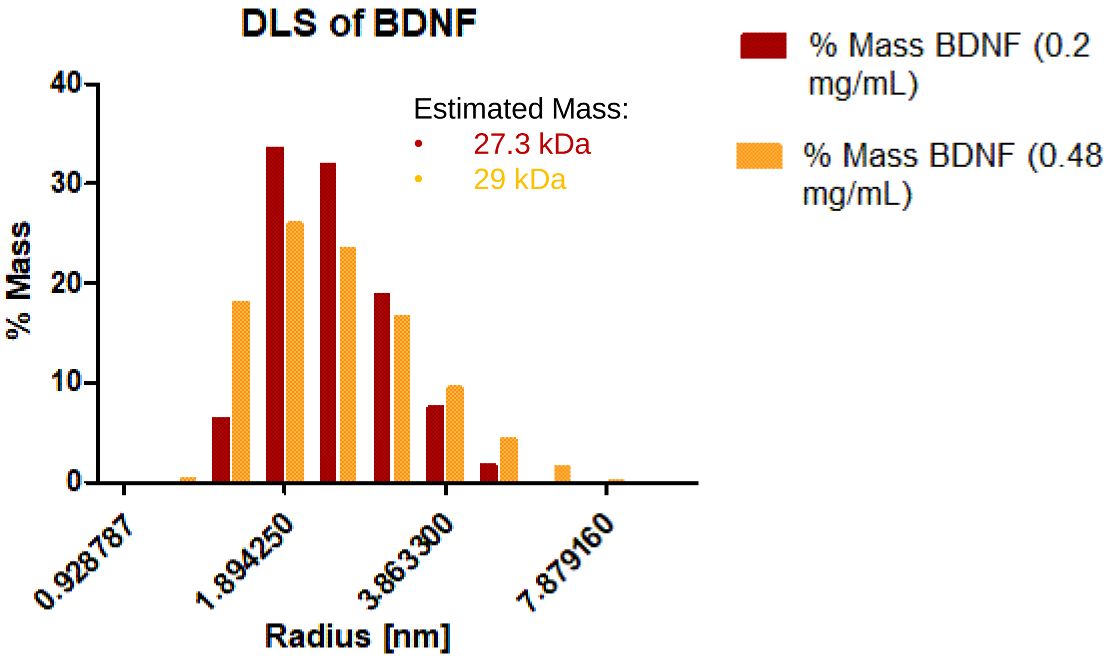

C: Dynamic light scattering revealed soluble BDNF to mostly be present in a dimeric state (theoretical monomer mass 14.5 kDa, dimer mass 29 kDa).

Figure S4: Complex binding behavior of TrkB with BDNF.

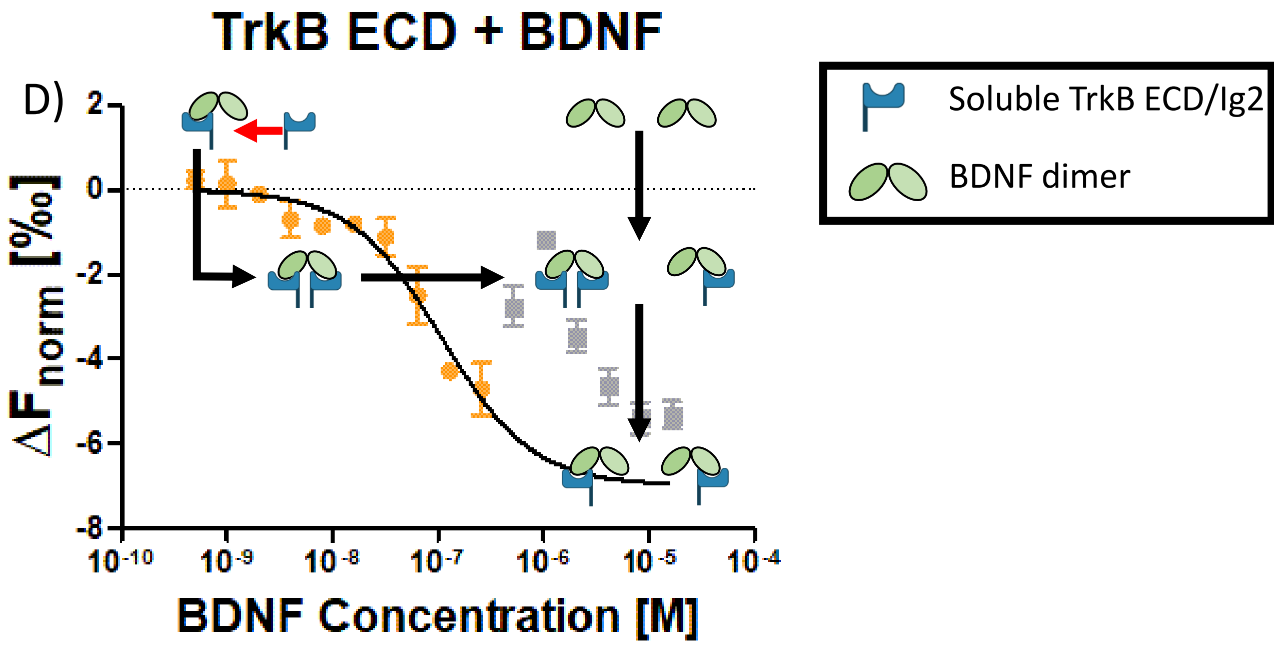

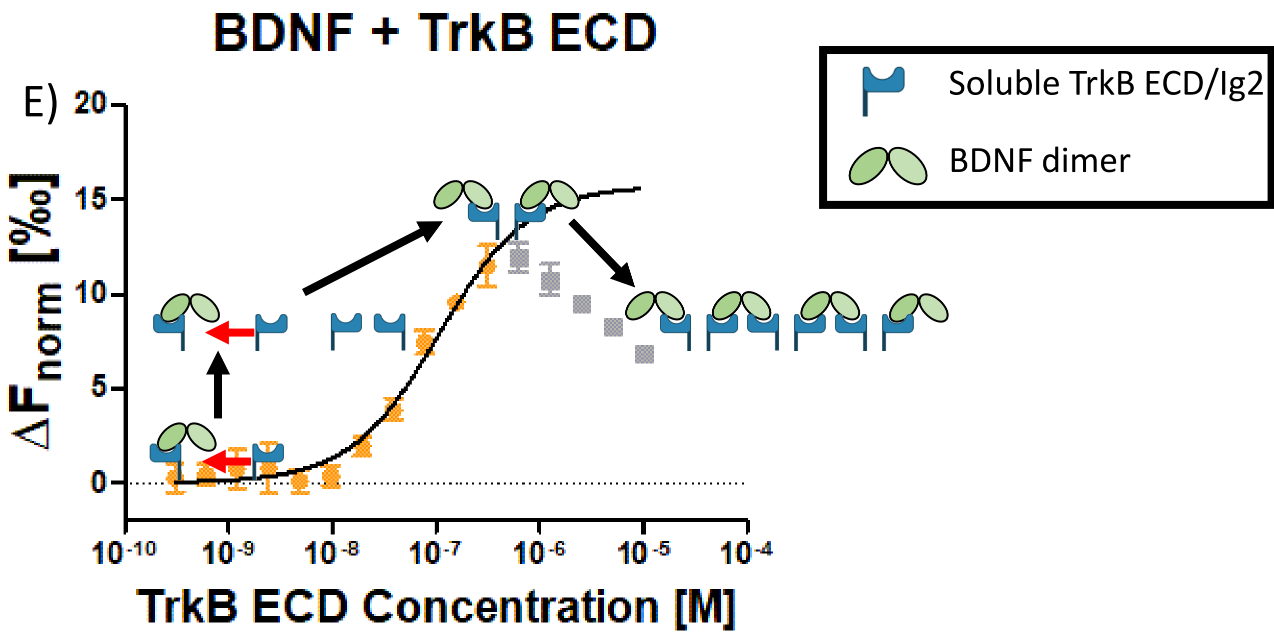

Schematic explanation for the mechanisms underlying the biphasic binding events observed during binding affinity measurements between TrkB and BDNF or vice versa.

D: Increasing BDNF concentrations in the dilution series with constant concentrations of TrkB ECD or Ig2 lead to an oversaturation of monomeric TrkB ECD or Ig2 with dimeric BDNF, effectively preventing the TrkB dimerization.

E: Increasing TrkB ECD or Ig2 concentrations in the dilution series with constant concentration of BDNF lead to BDNF-independent TrkB dimerization, allowing for BDNF binding in a mono-valent manner and ultimately multimerization of the BDNF-TrkB system.

Figure S5: Possible role of TrkB binding of proBDNF for the maturation of proBDNF.

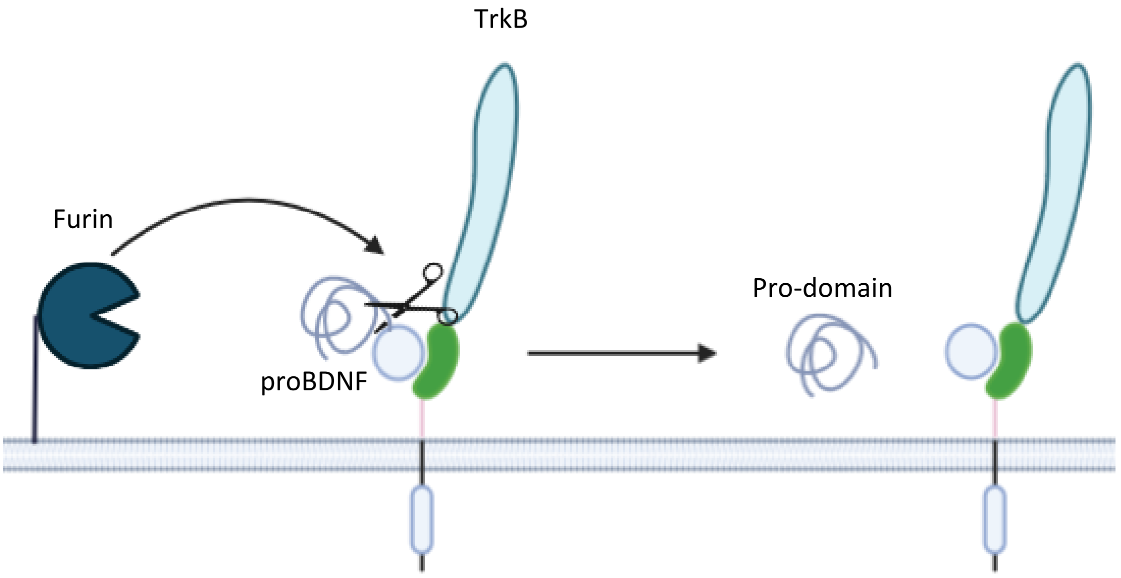

proBDNF binding to membrane-anchored TrkB may assist its maturation by membrane-bound activators (furin, plasmin).

Table S1: Chromatographic setup

chromatography	intact Ig2. legumain-digested Ig2, peptide and disulfide mapping of Ig2	intact pBDNF	peptide and disulfide mapping digested pBDNF
instrument	UltiMate™ U3000 RSLC, Thermo Fisher Scientific, Germering, Germany	Vanquish HPLC-system, Thermo Fisher Scientific, Germering, Germany	UltiMate™ U3000 RSLCnano, Thermo Fisher Scientific, Germering, Germany
autosampler temperature [°C]	4,0	4,0	4,0
injection type	in-line split-loop mode, 10 µL	5,0 µL	full loop, 1 µL
column name	Discovery BIO wide pore C18 column (Supelco, Bellefonte, PE, USA)	Discovery BIO wide pore C18 column (Supelco, Bellefonte, PE, USA)	Acclaim PepMap® RSLC column (Thermo Scientific™)
column chemistry	C18	C18	C18
column dimensions	150 × 2.1mm i.d., 3.0 µm particle size, 300 Å pore size	150 × 2.1mm i.d., 3.0 µm particle size, 300 Å pore size	15 cm x 300 µm i.d., 2.0 µm particle size, 100 Å pore size
flow rate [$\mu L.min^{-1}$]	200,0	100,0	1,2
column oven temperature [°C]	70,0	60,0	50,0
mobile phase A	H_2O + 0.10% FA	H_2O + 0.10% FA	H_2O + 0.10% FA
mobile phase B	ACN + 0.10% FA	ACN + 0.10% FA	ACN + 0.10% FA
linear gradient	5.0% B for 10.0 min, 5.0% – 40.0% B for 20 min, 40.0% – 80.0% B for 5.0 min, 80.0% B for 5.0 min and 5.0% B for 20 min	15.0% B for 3.0 min, 15.0% - 90.0% B for 27.0 min, 90.0% - 99.0% B for 0.1 min, 99.0% B for 5.0 min, 99.0% - 5.0% B for 0.1 min, 5.0% B for 5.0 min	1.0% B for 5.0 min, 1.0% - 30% B for 30.0 min, 30.0% - 60.0% B for 5.0 min, 99.0% B for 5.0 min, 1.0% B for 10.0 min
UV-detection [nm]	214	n.a.	n.a.
flow cell	1.4 µL	n.a.	n.a.

Table S2: Mass spectrometric setup

mass spectrometry	Intact Ig2	legumain-digested Ig2	intact and legumain-digested Ig2	peptide and disulfide mapping TrkB Ig2	intact pBDNF	peptide and disulfide mapping digested pBDNF
mass spectrometer	Q-Exactive (quadrupole-orbitrap)				Q-Exactive Plus (quadrupole-orbitrap)	
ion source	HESI	HESI	HESI	HESI	HESI	NSI
polarity	positive	positive	positive	positive	positive	positive
source heater temperature [°C]	250	250	250	250	150	-
spray voltage [kV]	3,5	3,5	3,5	3,5	4,5	1,5
sheat gas flow	30	30	30	30	10	0
auxiliary gas	10	10	10	10	8	0
spare gas	0	0	0	0	0	0
method type	full MS	full MS	AIF	Full MS-dd top 5 HCD	full MS	Full MS-dd top 10 HCD
total run time [min]	10.0 - 55.0	10.0 - 55.1	10.0 - 55.0	10.0 - 55.0	0.0 - 50.0	0.0 - 55.0
m/z scan range (full MS / MS^2)	500–3,000	500–3,000	400–2,500	400–2,000	1,000–3,500	400–3,000 (MS) / 200–2,000 (MS^2)
resolution (full MS / MS^2) at 200 m/z	17.500	140.000	140.000	70,000 (MS) / 17,500 (MS^2)	140.000	70,000 (MS) / 17,500 (MS^2)
AGC (full MS / MS^2)	3e6	3e7	3e6	3e6 (MS) / 5e5 (MS^2)	3e6	3e6 (MS) / 1e5 (MS^2)
max injection time (full MS / MS^2) [ms]	200	200	200	100 (MS) / 200 (MS^2)	150	100 (MS) / 50 (MS^2)
microscans	10	10	10	1	10	1
capillary temperature [°C]	320	320	320	320	300	250
S-lens RF level	80	80	80	80	50	60
in-source CID [eV]	0,0	0,0	0,0	0,0	0,0	0,0
NCE [%]	n.a.	n.a.	22	30	n.a.	28
dynamic exclusion [s]	n.a.	n.a.	n.a.	10,0	n.a.	10,0

Table S3: Mass spectrometric data evaluation

	disulfide mapping pBDNF
software	Byonics
manufacturer	Protein Metrics Inc.
sample digestion parameters	
cleavage site	KR
cleavage side	C-terminal
digestion specificity	fully specific
missed cleavages	2
instrument parameters	
precursor mass tolerance [ppm]	20
fragmentation type	CID low energy
fragment mass tolerance [ppm]	20
S-S, Xlink	disulfide / -2.015650 Da @ C
spectrum and protein output options	
charge state	n.a.
maximum precursor mass	10000
false discovery rate [%]	1
	intact pBDNF
algorithm	Xtract
source spectra method	average over selected retention time
retention time range [min]	22.0 -24.7
output mass range [Da]	1000 - 60000
sequence matching mass tolerance [ppm]	20
mass tolerance [ppm]	10

Table S4: TrkB cleavage assay

examplary for these protein stocks and final conentrations of 1 mg/ml TrkB ECD/Ig2 and 0.055 mg/ml legumain

	Cleavage Assay	TrkB Control	legumain Control
pH	7	7	7
buffer (µl)	31,13	32,50	43,62
pH stock (µl)	5,00	5,00	5,00
TrkB variant* (µl)	12,50	12,50	
legumain (µl)	1,38		1,38
final volume	50,00	50,00	50,00

--> incubate at RT for 10 mins (--> stop reaction with 50 µL 1 M Ammonium Acetate pH 8.6)

*TrkB ECD (29-428) or TrkB Ig2 (283-428)

pH stock:
1 M Tris/HEPES pH7 or 1 M citric acid pH 5.5

protein stocks:

TrkB ECD	4	mg/ml
TrkB Ig2	4	mg/ml
legumain	2	mg/ml

final concentration TrkB ECD/Ig2:	0,5-1	mg/ml
final concentration legumain:	0,007-0,055	mg/ml

buffer:
20 mM citric acid pH 5.5
50 mM NaCl
